# Design of an Acoustic Target Intrusion Detection System Based on Small-Aperture Microphone Array

**DOI:** 10.3390/s17030514

**Published:** 2017-03-04

**Authors:** Xingshui Zu, Feng Guo, Jingchang Huang, Qin Zhao, Huawei Liu, Baoqing Li, Xiaobing Yuan

**Affiliations:** 1 Science and Technology on Microsystem Laboratory, Shanghai Institute of Microsystem and Information Technology, Chinese Academy of Sciences, Shanghai 201800, China; zuxs@mail.sim.ac.cn (X.Z.); GuoFeng@mail.sim.ac.cn (F.G.); qinzhao21@mail.sim.ac.cn (Q.Z.); liuhw@mail.ustc.edu.cn (H.L.); sinowsn@mail.sim.ac.cn (X.Y.); 2 University of Chinese Academy of Sciences, Beijing 100049, China; 3 IBM-Research China Lab, Shanghai 201203, China; jchhuang@mail.ustc.edu.cn

**Keywords:** intrusion detection, small-aperture microphone array, wind noise, time delay estimation, WSN

## Abstract

Automated surveillance of remote locations in a wireless sensor network is dominated by the detection algorithm because actual intrusions in such locations are a rare event. Therefore, a detection method with low power consumption is crucial for persistent surveillance to ensure longevity of the sensor networks. A simple and effective two-stage algorithm composed of energy detector (ED) and delay detector (DD) with all its operations in time-domain using small-aperture microphone array (SAMA) is proposed. The algorithm analyzes the quite different velocities between wind noise and sound waves to improve the detection capability of ED in the surveillance area. Experiments in four different fields with three types of vehicles show that the algorithm is robust to wind noise and the probability of detection and false alarm are 96.67% and 2.857%, respectively.

## 1. Introduction

Real-time intrusion detection is really important in many mission-critical wireless sensor network (WSN) applications such as battlefield monitoring and security surveillance [1]. The sensors are randomly deployed in the complicated and time-varying military district. Hence, effective intrusion detection is a great challenge because of the processing capability and battery life resource limitations of sensor nodes that make up most economically viable WSNs [2]. Acoustic sensor has the advantages of low cost, low power consumption, and non-line of sight measurement which is widely adopted for a long time military terrain reconnaissance [3,4,5,6,7,8,9].

Target detection using acoustic time-domain signal is challenging as the signals are inevitably contaminated by wind noise and easily disturbed by road conditions, terrain, multipath, etc. [4]. Therefore, features employed by researchers are normally extracted from frequency domain [3,5] and time-frequency domain [6]. The harmonic line association (HLA) feature was utilized in [7,8] while it is difficult to extract HLA feature due to the non-stationary and wideband characteristic of vehicle acoustic signals. A wavelet-based algorithm was introduced in [9] but it is not suitable for real-time vehicle detection because it requires an intensive amount of computation and a large number of samplings.

These methods are well-suited to some geological environments where it can be assumed that the noise is stationary or slowly varying perform well. However, they pay little attention to wind noise, a particular type of noise in wild reconnaissance, which is hard to handle in the invasion detection application. Wind noise is generated by the pressure variations from flow turbulence deflect the microphone diaphragm [10]. Therefore, the wind turbulence between microphones is comparatively uncorrelated, which can be used to distinguish the sound waves from the wind noise [11,12]. A min peak correlation coefficient measure was designed in [12] for wind noise analysis using large aperture array with radius of one meter. Yet the low correlation is not a reliable feature for non-stationary wind noise detection with SAMA [10]. Therefore, the $\chi^{2}$ algorithm to robust wind noise detection was introduced in [10] for audio devices such as hearing aids, cochlear implants, phones and headsets. However, few researches exploiting the delay time between SAMA sensors to detect the wind noise in surveillance sensor except for calculating the velocity of sound [13] and wind [14]. This paper introduces a novel two-stage algorithm to reduce the high false alarm rate that is often caused by wind noise. The algorithm operates efficiently in the time domain and makes use of the time delay between the noise source reaching individual SAMA microphones.

In this paper, wind noise is mainly considered and effectively handled based on the considerable velocity divergences between wind noise and sound waves by a 4-element MEMS microphone array (MA) [15]. A two-stage detection algorithm composed of ED and DD is constructed with all its operations only in time-domain. The receiver operating characteristic (ROC) curves are used to evaluate the performance of the detector tested with cars, trucks and tracked vehicles in four different sites during the year 2013∼2015.

The rest of the paper is organized as follows: Section 2 describes the schematic of the intrusion detection algorithm energy delay detector (EDD); Section 3 describes the experiments conducted in four fields with three types of vehicles; The performance of the EDD is presented in Section 4; and the paper is concluded in Section 5.

## 2. The Schematic of Intrusion Detection Algorithm

Since we use numerous acronyms throughout this paper, we first list their full descriptions in Table 1.

In the remote area, the intrusion targets of interest are vehicles (including wheeled and tracked vehicles) and aircraft. Intrusion detection using an acoustic sensor is a challenging problem as the signals are inevitably contaminated by wind noise which is a common and inevitable disturbance. Wind noise always causes false alarm and is hardly to deal with because it is highly non-stationary and its power and spectral characteristics vary greatly. Since wind noise has a low velocity far less than that of sound waves, well designed time delay estimation (TDE) algorithm named DD should be able to differentiate the two phenomena.

**Figure 1:**
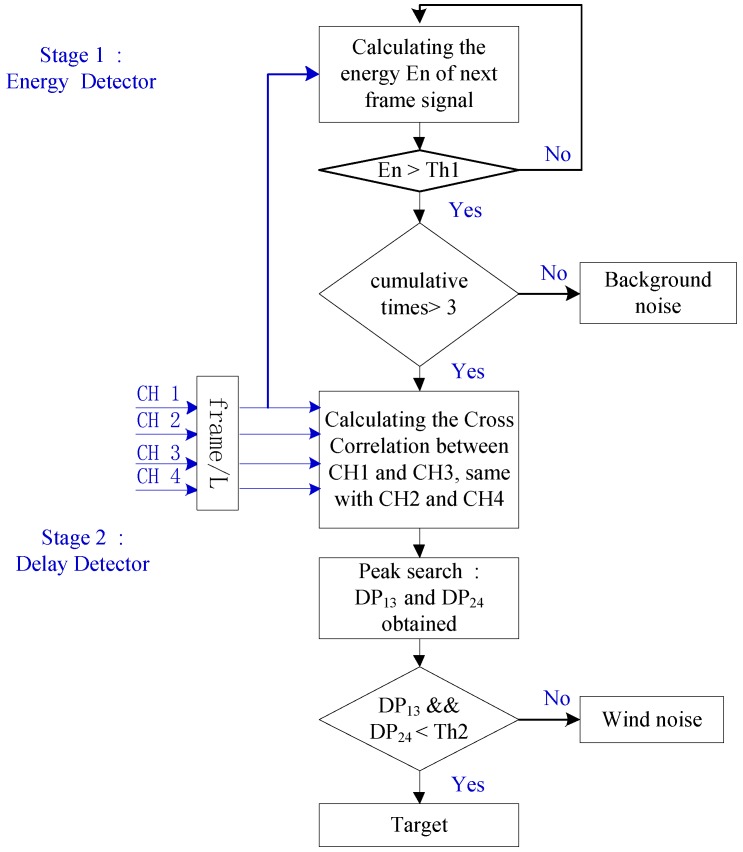
Block diagram of the proposed energy delay detector (EDD) ( Frame/L denotes dealing L points every frame, Th1 and Th2 denote the threshold of the first and second stage, CH1-4 means the four channel signals collected by 4-element microphone array, DP${}_{13}$ and DP${}_{24}$ denotes the delay point between channels).

On the other hand, the delay time of background noise between microphones is comparable to the delay time of sound. We should emphasize that the background noise includes but not limited to circuit noise and environmental sound noise except wind noise. Then, ED is utilized to deal with the background noise as the assistant of DD. Moreover, to avoid some other false alarm caused by short-term interference noise and animals in the field, we add the parameter cumulative times after the ED. Finally, a two-stage detection mechanism as shown in Figure 1 is constructed for reconnaissancing the remote terrain.

### 2.1. Energy Detector

The ED measures the energy associated with the received signal over a specified time duration and bandwidth [16]. The measured value is then compared with an appropriately selected threshold to determine the presence or absence of the target signal. A predefined threshold is required, which determines the performance of the detector, including the probability of detection (PD), the probability of false alarm (PFA), and detecting distance. Since missing the detection of the target will lead to a great loss in military applications, it is crucial for selecting an appropriate threshold to satisfy the requirement of PD.

A common ED can be expressed as Equation (1).

(1) $$E_{n}=\sum_{i=1}^{L}x_{n}^{2}\left(i\right)$$

where *i* = 1,...,*L* and $x_{n}\left(i\right)$ denotes the *n*th frame of *i*th point, *L* denotes the length of frame.

In order to reduce computational complexity, we replace Equation (1) with Equation (2).

(2) $$E_{n}=\frac{1}{L}\sum_{i=1}^{L}\left|x_{n}\left(i\right)\right|$$

### 2.2. Time Delay Estimation

The time delay between two channel signals at a MA has been proven to be a useful parameter. Signal enhancement, target localization and recognition are some applications of TDE. We have implemented various methods for the TDE as described in Table 2, including basic cross correlation (BCC) and generalised cross correlation (GCC). The main difference between the two algorithms is that GCC uses the weighting functions shown in Table 3 to improve the performance of TDE [17]. As the distinct delay time between wind noises and sound waves, slightly improving the accuracy of TDE is nonsignificant to our experiment but increasing the power consumption. Therefore, we employ the simplest BCC method in this paper due to the limited power and processing resources in sensors.

In Table 2, $R_{s1s2}\left(\tau \right)$ is the cross correlation of two channel signals, and $\tau_{12}$ is the obtained delay time between two channel signal. More specific description of the expressions in Table 2 and Table 3 can be found in [17].

### 2.3. Energy Delay Detector

Although ED is simple and has a high PD, it is vulnerable to wind noise and at its threshold it is difficult to have a good tradeoff between the PD and PFA. Utilizing the difference of propagation velocity between wind noise and sound waves, we can reject wind noise robustly by assessing the delay point (DP) between MA. DP denotes delay point between channels. More specifically, DP equals delay time multiplied by sampling frequency. For example, DP${}_{13}$ denotes the DP between CH1 and CH3, it equals the delay time between CH1 and CH3 multiplied by sampling frequency. This method can efficiently distinguish the target from wind noise even in low signal-to-noise ratio (SNR), because wind noise moves much slower than sound waves even in large wind scale.

We exclude the wind noise through calculating the DP between the different channels. Unfortunately, this does not solve the problem of distinguishing other sources of environmental noise because background noise has virtually the same DP as sound waves. Therefore, a two-stage detector named EDD is proposed as shown in Figure 1. The first stage is the ED for finding out the anomalous situation from the background noise, and the second stage is the DD for ascertaining that the anomalous case is a target or wind noise. Although the two components of EDD are simple and commonly used in different fields, the synergetic combination of them in this area is unique and is very well suited to resource-limited unattended sensors.

## 3. Experimental Description

**Figure 2:**
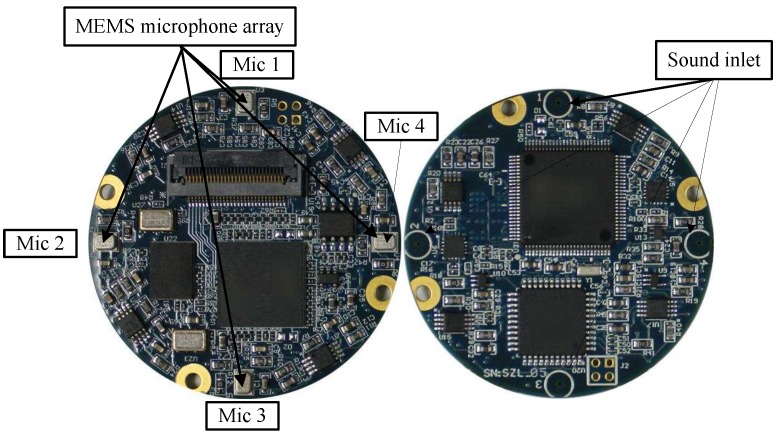
Photograph of the mall-aperture microphone array (SAMA) system and the array diameter is 4 cm.

**Figure 3:**
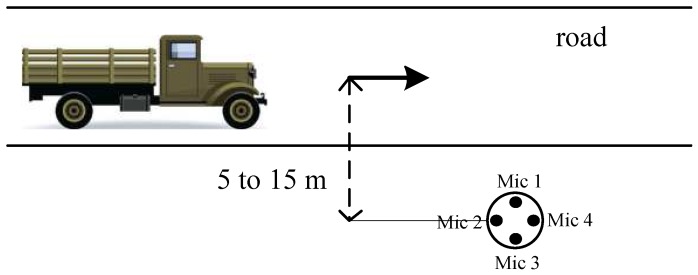
Layout of the experimental scenario.

We designed some experiments to empirically evaluate the effectiveness of the EDD method which utilized a single uniform circular array (UCA). The UCA had a diameter of *d* = 0.04 m and was composed of four ADMP504 MEMS microphone (Analog Devices, Norwood, MA, USA) as shown in Figure 2. The inputs of these microphones were fed into separate channels of a 4-channel 16-bit simultaneous ADC (MAXIM MAX11043) which was sampled at a rate of 8192 Hz [15]. The sensors were placed 5 to 15 m away from the road as shown in Figure 3 and wind scale is recorded by ultrasonic anemometer at the same site during the experiments.

**Figure 4:**
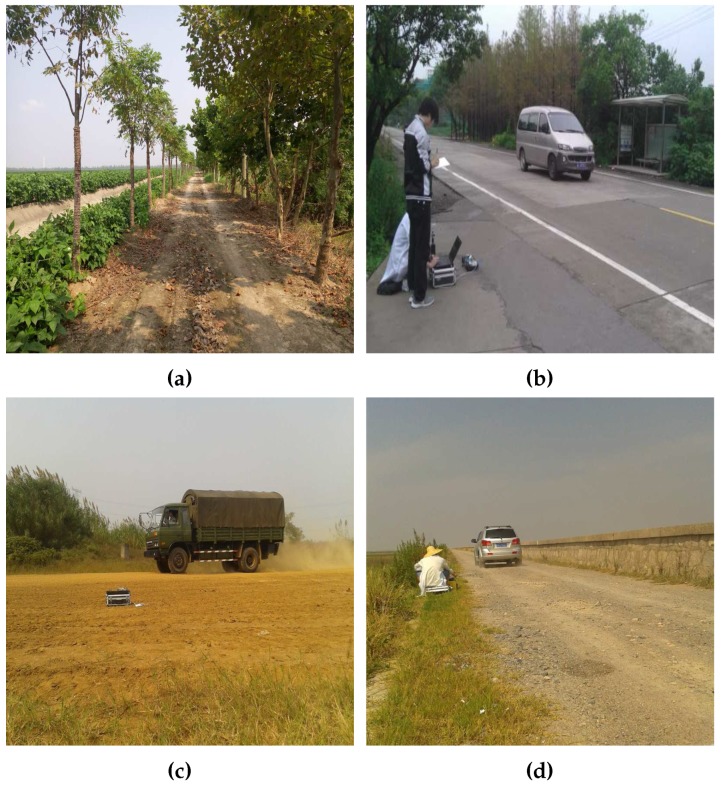
Four different experimental environments in Nanjing, Anhui and Shanghai. (**a**) dirt road; (**b**) concrete road; (**c**) mud road; (**d**) gravel road.

Experimental studies were performed from June 2013 to December 2015 on Chongming Island, Zhoushan Island, Nanjing and a suburban district around Shanghai where the wind power level is usually less than 6. The four experimental environments are shown in Figure 4 and the compositions of our sample set are shown in Table 4. The application of windshield to prevent the noise generation acoustically is widely adopted in field experiments. While the devices used in WSN are getting smaller to save more space, we did not use wind-shelters on the microphones during these experiments.

## 4. Evaluations

### 4.1. Detection of Wind Noise and Vehicle

Wind noise is generated by the pressure variations from flow turbulence deflect the microphone diaphragm [10]. Therefore, we presume that wind noise is the laminar model for simplicity. In our case, *d* = 0.04 m, *f* = 8192 Hz, and $v_{sound}\approx 340$ m/s. The DP of the sound wave is approximately 1 referring to Equation (3).

(3) $$DP=\frac{d}{v}*f$$

However, we find 2 is the most applicable threshold separating the wind noise with sound waves through our experiment because of the inaccurate wind noise model. The DP of wind noise is greater than 20 according to Equation (3) when the speed of wind is less than 16 m/s. Therefore, it easily distinguishes the wind noise and not result in false target.

**Figure 5:**
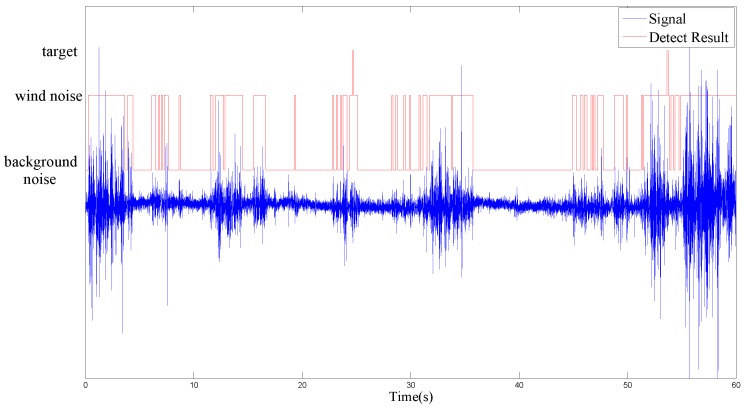
Result of EDD in the scene of no intrusion target.

**Figure 6:**
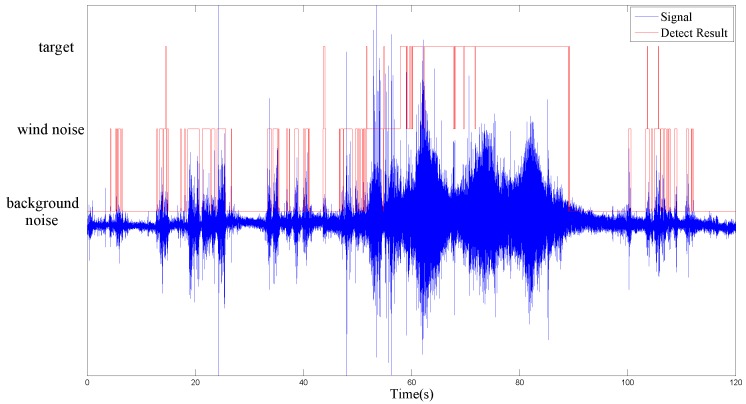
Result of EDD in the scene of 3 cars with 100 m interval in 120 s signals.

Figure 5 and Figure 6 show the detection capability of the EDD in the two experimental scenes of no intrusion target and 3 cars with 100m interval, respectively. Generally, the task of intrusion detection is distinguishing between target and no target. However, for clearly demonstrating the algorithm’s ability of overcoming the false alarm caused by wind noise, we divide the situation of target into target and wind noise. Hence, all the frames labeled with wind noise in Figure 5 and Figure 6 would result in false alarms without the application of the second stage DD.

The experimental results indicate that EDD can distinguish wind noise precisely which leads to the majority of the false alarms in the remote surveillance area. For clearly demonstrating the detection capability of the EDD frame by frame, the cumulative times on the first stage is 1 which is inconsistent with the actual experiment employed 3. Therefore, the false detected targets in Figure 6 can be eliminated in practical application.

### 4.2. Experimental Results

**Figure 7:**
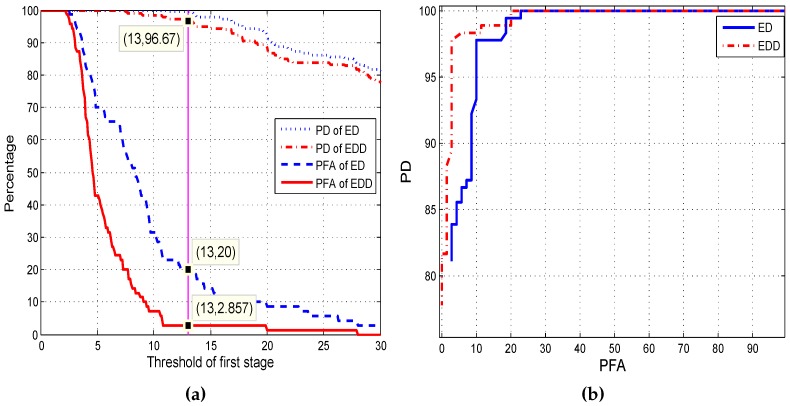
Comparing the capability of ED and EDD. (**a**) PD and PFA of ED and EDD; (**b**) ROC curves of ED and EDD.

Th1 is the empirical energy threshold of the first stage and Th2 is the DP threshold of the second stage. In Figure 7a, as conditions of two Threshold levels Th1 and Th2, Th1 is varied from 0 to 30, Th2 is fixed 2, ROC curves describing the relationship between PD, PFA and Th1 are obtained. Comparing the PD and PFA curves of ED and EDD in Figure 7a, ED can suppress the false alarm mostly (PD: 99.44%, PFA: 20%), choosing 13 and 2 as the threshold of the two-stage detector, respectively. The probability of triggering the second stage is 20% indicating that the detection system runs the lower power consumed first stage algorithm in 80% of the reconnaissance time. As expected, slight degradation in PD is obtained because of the employing of the second stage TDE algorithm. Generally speaking, the reducing of PFA is always along with the decreasing of PD.

**Figure 8:**
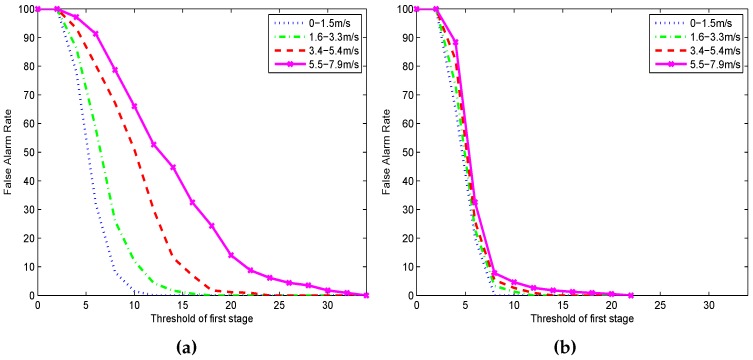
Comparing the PFA of (**a**) ED; and (**b**) EDD under four different wind scale.

The performance of the ED and EDD under different wind scales is shown in Figure 8. Results indicate the EDD is robust to wind noise as the PFA curve without obvious change along with the great change of wind scale. We should emphasize that there have no ROC curves because only wind noise samples are employed to evaluate the EDD in the capability of reducing PFA. The ED has the advantages of high PD and larger detection range, while has the weakness of high PFA at the same time. The EDD, inheriting the advantages of ED and decreasing PFA from 20% to 2.857%, has significant well detection ability evaluated in four diverse fields Figure 7b. On the other hand, low pass filter can be used to preprocess the occasion when wind noise is so large that its amplitude is greater than that of target signals. It is effective because the energy of the wind noise is mainly in low frequency.

**Figure 9:**
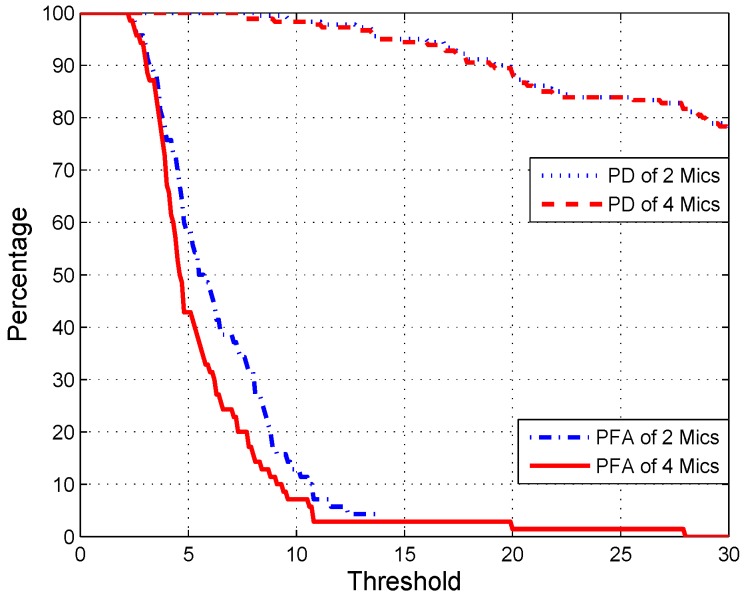
Comparing the performance of EDD using two and four microphones.

Furthermore, we also can utilize only two microphones to compliment the detection mission which is commonly utilized in hearing aids. Nevertheless, the PFA is slightly higher than that of the four microphones as shown in Figure 9 because no DP between two microphones when the wind direction is exactly perpendicular to the line between the two microphones. On the other hand, the PD curves of 2 Mics and 4 Mics in Figure 9 almost overlapped with each other, which indicates that our algorithm has a good capability to suppress PFA.

## 5. Conclusions

We presented a simple and effective two-stage intrusion detection mechanism using SAMA which is evaluated in four diverse terrains with three types of vehicles. The EDD conquers the high PFA weakness of the ED while conserving the merit of high PD. Furthermore, all its operations are in time-domains so that they have ultra low computational complexity and power consumption. Although the components of EDD are simple and commonly used in different fields, their synergetic combination has been empirically shown to be effective in mitigating the effect of wind noise and they can be implemented in a limited resource unattended sensor environment. The ROC curves show the PFA decreased from 20% to 2.857% reached by EDD in comparison with ED. Experimental results under four wind scales also show the robustness of the algorithm. The algorithm is mainly designed for intrusion detection in mission-critical WSN. However, the algorithm could also provide a reference for other applications such as wind noise detection in hearing aids.

## Figures and Tables

**Table 1:** Acronyms and their full descriptions.

Acronym	Full Description
ED	Energy Detector
DD	Delay Detector
EDD	Energy Delay Detector
MA	Microphone Array
SAMA	Small-Aperture Microphone Array
WSN	Wireless Sensor Network
ROC	Receiver Operating Characteristic
TDE	Time Delay Estimation
PD	Probability of Detection
PFA	Probability of False Alarm
BCC	Basic Cross correlation
GCC	Generalised Cross Correlation
SNR	Signal-to-Noise Ratio
UCA	Uniform Circular Arrays

**Table 2:** The mathematical expressions of basic cross correlation (BCC) and generalised cross correlation (GCC) method.

TDE	Mathematical Expression $R_{s_{1}s_{2}}(\tau )$	Estimated Time Delay $\tau_{12}$
BCC	$1/(T-\tau )\int_{\tau }^{T}s_{1}\left(t\right)s_{2}(t-\tau )\mathrm{dt}$	$\arg\max_{\tau }R_{s_{1}s_{2}}(\tau )$
GCC	$\int_{-\infty }^{\infty }W(\omega )S_{1}(\omega )S_{2}(\omega )e^{i\omega \tau }d\omega$		

**Table 3:** Weighing functions adopted in the GCC method [17].

Method Name	Weighting Function W(x)
Cross correlation	1
Roth Impulse response	$1/G_{s1s1}\left(f\right)$
Phase transform	$1/|G_{s1s2}\left(f\right)|$
Smoothed coherence transform	$1/\sqrt{G_{s1s1}\left(f\right)G_{s2s2}\left(f\right)}$
Eckart filter	$G_{s1s1}\left(f\right)/\left[G_{n1n1}\left(f\right)G_{n2n2}\left(f\right)\right]$
Maximum likelihood	$|\gamma_{12}{\left(f\right)}^{2}\left|/\right|G_{s1s2}\left(f\right)|[1-|\gamma_{12}{\left(f\right)}^{2}\left|\right]$

**Table 4:** Sample sets collected in four different experimental fields, every sample is 60 s with sampling rate 8192 Hz.

Geology	Road Type	Car	Truck	Tracked Vehicle	Noise
Chongming	dirt road	13	15	19	34
Zhoushan	concrete road	17	14	0	33
Fengxian	sand road	15	12	18	35
Nanjing	mud road	18	18	22	38
Total Runs(mins)	320	63	59	58	140
